# Removal of Cardiopulmonary Resuscitation Artifacts with an Enhanced Adaptive Filtering Method: An Experimental Trial

**DOI:** 10.1155/2014/140438

**Published:** 2014-03-27

**Authors:** Yushun Gong, Tao Yu, Bihua Chen, Mi He, Yongqin Li

**Affiliations:** ^1^School of Biomedical Engineering, Third Military Medical University and Chongqing University, 30 Gaotanyan Main Street, Chongqing 400038, China; ^2^Emergency Department, Sun Yat-Sen Memorial Hospital of Sun Yat-Sen University, Guangzhou 510120, China; ^3^Institute of Cardiopulmonary Cerebral Resuscitation, Sun Yat-Sen University, Guangzhou 510120, China

## Abstract

Current automated external defibrillators mandate interruptions of chest compression to avoid the effect of artifacts produced by CPR for reliable rhythm analyses. But even seconds of interruption of chest compression during CPR adversely affects the rate of restoration of spontaneous circulation and survival. Numerous digital signal processing techniques have been developed to remove the artifacts or interpret the corrupted ECG with promising result, but the performance is still inadequate, especially for nonshockable rhythms. In the present study, we suppressed the CPR artifacts with an enhanced adaptive filtering method. The performance of the method was evaluated by comparing the sensitivity and specificity for shockable rhythm detection before and after filtering the CPR corrupted ECG signals. The dataset comprised 283 segments of shockable and 280 segments of nonshockable ECG signals during CPR recorded from 22 adult pigs that experienced prolonged cardiac arrest. For the unfiltered signals, the sensitivity and specificity were 99.3% and 46.8%, respectively. After filtering, a sensitivity of 93.3% and a specificity of 96.0% were achieved. This animal trial demonstrated that the enhanced adaptive filtering method could significantly improve the detection of nonshockable rhythms without compromising the ability to detect a shockable rhythm during uninterrupted CPR.

## 1. Introduction

Early defibrillation is critical for the survival of patient who suffered from cardiac arrest [1, 2]. However, the application of high quality of cardiopulmonary resuscitation (CPR) introduces strong artifact components into the electrocardiogram (ECG) signal, which reduces the accuracy of the shock/nonshock decision of automated external defibrillators (AEDs) [3]. Thus, chest compressions (CC) are mandated to be interrupted in the current AEDs in order to perform a reliable rhythm analysis and provide appropriate defibrillation prompt to the rescuers. But even seconds of interruptions of CC adversely affects the rate of restoration of spontaneous circulation (ROSC) and survival [4]. According to an experimental study, the likelihood of successful resuscitation decreased as much as 50% with a 20-second interruption of CC [5]. Actually, clinical studies have also confirmed that longer pauses in CC before and after defibrillator shocks were independently associated with a decrease in survival to hospital discharge [6, 7]. When the hands-off intervals were minimized, significantly better outcomes were achieved and reported [8, 9]. Therefore, the latest guidelines from the American Heart Association (AHA) and the European Resuscitation Council (ERC) recommended minimizing these hands-off intervals between compression and shock [10, 11].

If accurate cardiac rhythm analysis can be performed during CPR, these interruptions will be minimized or totally avoided. During the last decade, numerous digital signal processing techniques have been developed to remove the artifacts or interpret CC corrupted ECG during CPR. Sensitivity and specificity are the proportion of correctly identified shockable and nonshockable rhythms, respectively, and are used to evaluate the performance of artifact suppression method. Algorithms removing artifacts using only the ECG signal, including independent component analysis (ICA) [12] and coherent line removal algorithm [13], have improved the sensitivity to 99.8% and the specificity to 83.2% for detecting a shockable rhythm. Methods filtering the CPR artifact using additional references, such as Gabor multipliers [14], Kalman filter [15], adaptive filter [16–19], and multichannel recursive adaptive matching pursuit (MC-RAMP) filter [20], have improved the sensitivity and specificity to 95.6% and 90.5%. To identify a shockable rhythm during CPR, Li et al. [21] searched the identifiable components directly in the corrupted ECG signal using morphology consistence evaluation. A sensitivity of 93.3% and specificity of 88.6% were reported in a dataset which consisted of 229 victims during out-of-hospital cardiac arrest. Although the sensitivity for detecting a shockable rhythm was significantly improved with the application of these techniques, the specificity was still below the 95% limit recommended by the AHA task force on AEDs for accurately detecting nonshockable rhythms [22]. Further studies are, therefore, still required to analyze the interaction between the artifact and underlying rhythms and to improve the accuracy of nonshockable rhythm decision [23, 24].

In the present study, the effects of CC on signal-to-noise ratio (SNR) at different types of underlying rhythms (ventricular fibrillation (VF), pulseless electrical activity (PEA), and asystole (ASY)) were firstly analyzed in an adult porcine model of prolonged cardiac arrest and CPR. An enhanced adaptive filtering method was then developed to suppress the CPR artifact and evaluated by comparing the sensitivity and specificity for shockable rhythm detection before and after filtering.

## 2. Materials and Methods

### 2.1. Experiment Procedure and Data Collection

The experimental data were collected from 22 male adult pigs that experienced prolonged cardiac arrest and CPR. The porcine model has been well established to simulate real out-of-hospital scenarios due to the fact that heart size, blood pressure, and heart rate are similar to those in humans [25]. Anesthesia was initiated by intramuscular injection of ketamine (20 mg/kg) and was completed by ear vein injection of sodium pentobarbital (30 mg/kg). VF was electrically induced by applying a 5 mA alternate current through a pacing catheter in the right ventricle. CPR, including CC and ventilation, was begun after 6 minutes of untreated VF (Group A) in 14 animals [26]. The compression depth (CD) was randomized to either 25% or 17% of the anterior posterior diameter of the chest during the first 4 minutes of CPR and 20–25% after 4 minutes. In another 8 animals with the same weight and chest size, CPR was begun after 11 minutes of untreated VF (Group B). CD was comparable to 20–25% of the anterior posterior diameter of the chest. For all of the animals, manual CC were performed by two experienced emergency medical doctors at a rate above 100 per minute. The animals were manually ventilated with a bag-valve device during CPR. CC were synchronized to provide a compression/ventilation ratio of 30 : 2 with equal compression-relaxation intervals. After 2 minutes of CC in Group A and 6 minutes of compression in Group B, a defibrillation was attempted with a single 120 J rectilinear biphasic shock. One dose of epinephrine (30 *μ*g·kg^−1^) was given through the right atrial catheter after 2 minutes of CPR in Group B. CC were immediately resumed followed by ECG rhythm analysis within 5 seconds until confirmation of spontaneous circulation. If spontaneous circulation was not restored, CC were continued for another 2 minutes, after which defibrillation was attempted with another single 120 J shock. This sequence was repeated for a maximum of 5 cycles.

The ECG, acceleration, and transthoracic impedance (TTI) waveform were continuously measured and recorded through a data acquisition system supported by Windaq hardware/software (Dataq Instruments Inc., Akron, OH, USA) at a sample rate of 300 Hz. During CC, the acceleration and TTI signals also served as feedback to control the compression rate and depth. The ECG was measured from the output of a commercial defibrillator (M-Series, Zoll medical corporation, Chelmsford, MA, USA) with the use of a hard gel type of adult defibrillation/pacing pads (stat-padz, Zoll Medical Corporation, Chelmsford, MA, USA) that were applied with an anterior to lateral placement. TTI waveform was recorded through a user designed circuit which was parallelly connected with the defibrillator using a sinusoid-wave excitation current of 2 mA and 30 kHz across the defibrillation pads. The acceleration signal was recorded from an accelerometer-based handheld CPR device (CPR-D-padz, Zoll Medical Corporation, Chelmsford, MA, USA) that was placed on the surface of the animal's chest just above the heart and underneath the rescuer's hands during CC.

**Figure 1:**
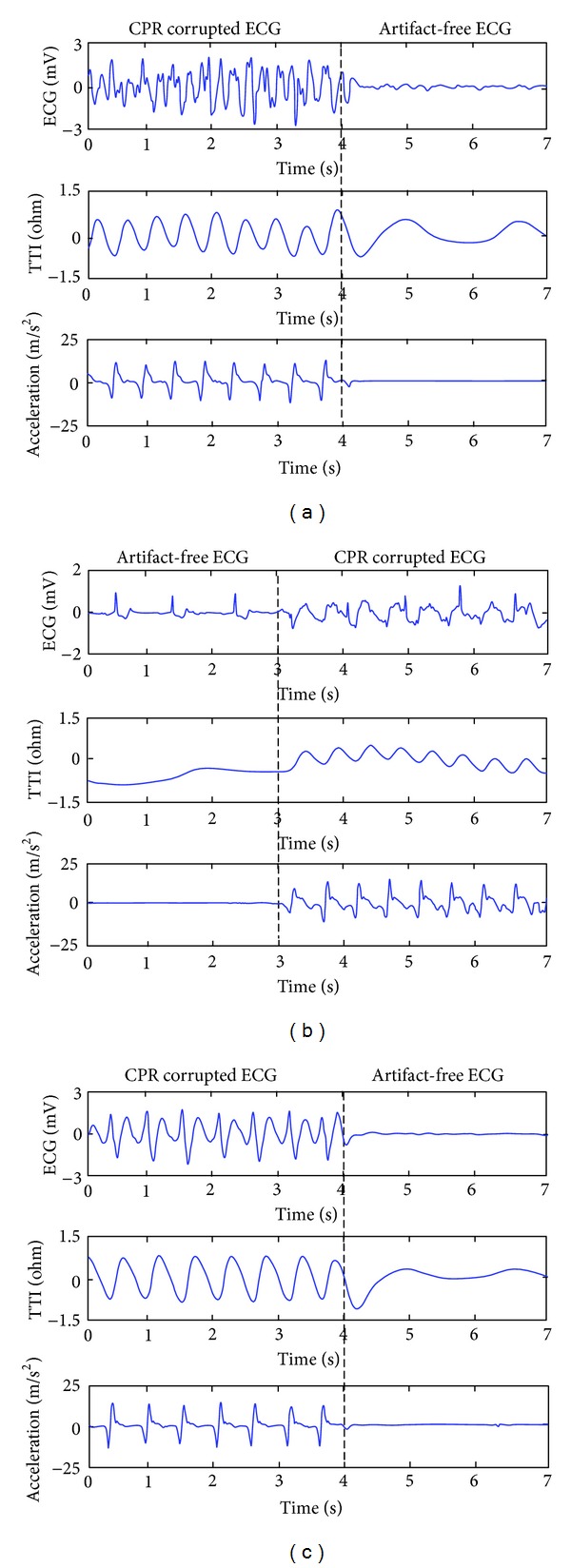
Segments of ECG and reference signals during cardiopulmonary resuscitation (CPR). (a) Ventricular fibrillation with and without chest compression (CC). (b) Pulseless electrical activity (PEA) without and with CC. (c) Asystole (ASY) with and without CC. TTI: transthoracic impedance.

Data were analyzed offline through user designed software using Matlab (The MathWorks, Inc., Natick, MA, USA). ECG, together with acceleration and TTI signals during CPR, was extracted and annotated from the digitalized experimental records. The CD was calculated from the double integration of acceleration signal. Each segment consisted of 4-second corrupted signal and 3-second artifact-free signal, either during ventilation or during rhythm analysis. These segments were then annotated as VF, PEA, or ASY by an experienced emergency medical doctor. As shown in Figure 1, a disordered electrical activity without the presence of observational QRS and with the peak-to-peak voltage greater than 0.1 mV was annotated as VF. The presence of at least one QRS complex in a segment was classified as PEA. A segment with peak-to-peak voltage less than 0.1 mV was annotated as ASY. Segments with rhythm transitions or defibrillation were excluded from the dataset.

### 2.2. Estimation of SNR

**Figure 2:**
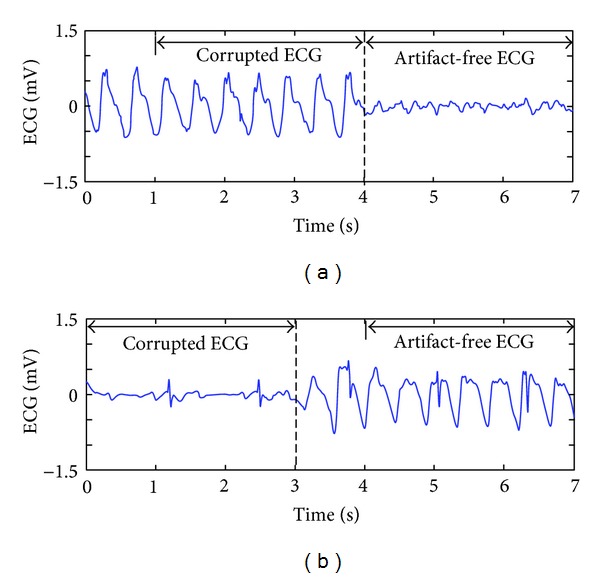
Examples of signal selection for SNR estimation. The CPR corrupted signal was selected either from the latest 3 seconds of chest compression (CC) (a) or 1 second after the beginning of CC (b).

To investigate the effects of CC on SNRo (before filtering) at different types of underlying rhythms (VF, PEA, and ASY) and performance of the proposed filtering method, we estimate the SNRo and the SNRf (after filtering) of the CPR corrupted ECG based on the contiguous artifact-free signal [27]. Assuming that the underlying ECG and CPR artifact are uncorrelated, the power of CPR artifact can be obtained through subtracting the power of corrupted ECG by the power of clean ECG.  Figure 2 shows the examples of signal selection for SNRo estimation in each segment. A 3-second corrupted ECG signal and another 3-second artifact-free signal are used to calculate the SNRo with the following equation:

(1) $$\begin{array}{c} \text{SNR}=10\cdot \text{lo}{\text{g}}_{10}\left(\frac{\sigma_{s}^{2}}{\sigma_{x}^{2}-\sigma_{s}^{2}}\right), \end{array}$$

where *σ*_*s*_^2^ is the variance of underlying ECG signal and *σ*_*x*_^2^ is the variance of corrupted ECG signal. The SNRf is also estimated with (1), except that the variance of underlying ECG is calculated by the filtered uncorrupted 3-second signal, and the variance of artifact is calculated by the subtraction of the variance of underlying ECG and the variance of filtered corrupted ECG signal.

The estimation is based on the hypothesis that time-limited VF and ASY can be considered quasi-stationary signal. On the other hand, since the energy of a normal sinus rhythm depends on the number of QRS complexes appearing within a segment, we therefore exclude the segments that have unequal numbers of QRS complex within the selected artifact-free and corrupted ECG signals when the underlying rhythm is annotated as PEA.

### 2.3. The Enhanced Adaptive Filtering Method

**Figure 3:**
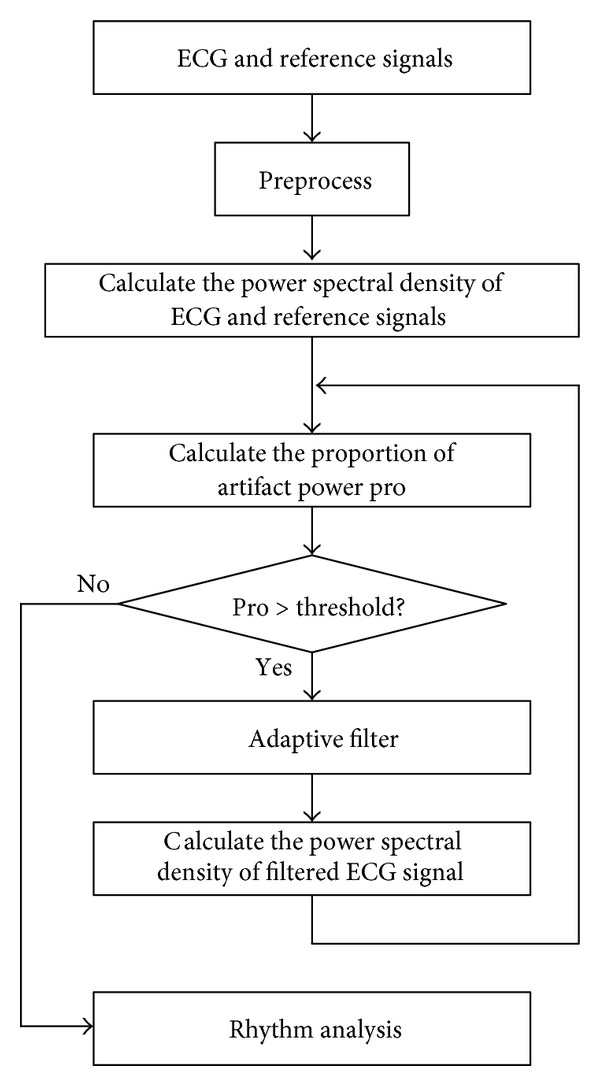
Flowchart of the enhanced adaptive filtering method.

To suppress the CC related artifacts (CC-artifact), an enhanced adaptive filtering method is developed by estimating the proportion of artifact within the CPR corrupted ECG signal. The flowchart of the proposed method is shown in Figure 3.

The corrupted ECG and reference (TTI) signals are firstly preprocessed by a 4th order Butterworth band-pass filter (0.2–45 Hz) to remove offset and high frequency noise. The power spectral density (PSD) of reference and preprocessed ECG signals are then calculated through dividing the square of the amplitude of fast Fourier transform (FFT) by the length of data points. The frequency of CC *f*_CC_ is obtained by the PSD of TTI:

(2) $$\begin{array}{c} f_{\text{CC}}=\arg\underset{f}{\max}\;P_{\text{TTI}}\left(f\right). \end{array}$$

The power of artifact is computed through the PSD of corrupted ECG with the use of *f*_CC_ and its harmonics. The proportion of the artifact power pro is calculated by

(3) $$\begin{array}{c} \text{pro}=\frac{\sum_{k=1}^{N}P_{S}\left(k\cdot f_{\text{CC}}\right)}{\sum_{f=0}^{f_{S}/2}P_{S}\left(f\right)}, \end{array}$$

where *k* is the order of harmonics (*N* = 3) and *f*_*s*_ is the sampling rate.

The proportion of the artifact power is then compared with a predefined threshold. If the proportion pro is greater than the preset threshold, the adaptive filter will be applied to the ECG signal to suppress the CPR artifact.

In this enhanced adaptive filtering method, normalized least mean squares (NLMS) is used to adjust the coefficient matrix of adaptive filter, and the step size is dynamically adjusted by the estimated artifact proportion pro:

(4) $$\begin{array}{c} W\left(n\right)=W\left(n-1\right)+\frac{\mu \cdot \text{pro}}{{||X||}^{2}}\cdot X\left(n-1\right)\cdot e\left(n\right). \end{array}$$

The step size *μ* is limited by the norm of reference signal ||*X*|| and proportion of artifact pro. The coefficient matrix *W*(*n*) at state *n* is decided by the previous state *W*(*n* − 1), the reference signal TTI *X*(*n* − 1), and the estimated ECG signal *e*(*n*):

(5) $$\begin{array}{c} e\left(n\right)=s_{\text{in}}\left(n\right)-W\left(n\right)X\left(n\right), \end{array}$$

where  *s*_in_(*n*) is the input corrupted ECG signal and *W*(*n*)*X*(*n*) is the estimated CPR artifact.

After filtering, the proportion of artifact pro of the filtered signal is recalculated to assess the SNRf level. If pro is still greater than the preset threshold, another iteration of filtering process will be applied to the filtered signal with updated step size. Otherwise, the filtered ECG signal will be outputted for rhythm analysis. In this study, the length of the coefficient *W*(*n*) is 21, and the step size *μ* is 0.15.

In order to compare the performance with the traditional fixed coefficient high-pass filter [28], a 4th order Butterworth high-pass filter is performed to the corrupted ECG signal to suppress the CPR artifact. Since the average compression rate is 2.11 Hz in this study, the cutoff frequency is 6.5 Hz to remove the first 3 harmonics of the artifact.

### 2.4. Rhythm Classification Algorithm

To evaluate the performance of the proposed method, the sensitivity and specificity for detecting a shockable rhythm before and after filtering are compared with an established rhythm classification algorithm named phase space reconstruction (RSR) [29, 30]. This specific algorithm is selected because it can provide accurate rhythm classification within a relative short time window. In this method, signal *s*(*t*) is plotted on *x*-axis and *s*(*t* + *τ*) with a delay time of *τ* is plotted on *y*-axis to form a two-dimensional phase space diagram. A 40 × 40 grid is produced and the number of boxes visited by the signal is counted. Ratio *r*′ is calculated through dividing the area that is filled with signal curve *B*_*v*_ by the total area of the diagram *B*_*a*_. In the current study, the maximum number of data points visited in the box *C*_max⁡_ is used to modify the ratio *r*′ which is used to classify PEA and VF:

(6) $$\begin{array}{c} r^{\prime }=\frac{B_{v}}{B_{a}}+\frac{1}{C_{\max}}. \end{array}$$

The average peak-to-peak amplitude of the filtered signal *A*′ is used to detect ASY. The 3- second ECG signal is split into 3 rectangular nonoverlapping windows. And the difference between maximum and minimum of the signal in each window is calculated and the average of these differences is represented as the value of *A*′.

A 3-second rectangular window is used to perform PSR, and the value of *τ* is 0.5 seconds. The threshold of the amplitude *A*′ and the ratio *r*′ are optimized with the artifact-free ECG signals to produce the optimum sensitivity/specificity values. The classification criteria are presented as

(7) A′≤0.1 mV       ASYA′>0.1 mV, r′≤0.24 PEAA′>0.1 mV, r′>0.24 VF.

### 2.5. Statistical Presentation

The distributions of SNRo of the CPR corrupted ECG signal did not pass the Kolmogorov-Smirnov normality test and were presented as medians (25/75 percentile). The Wilcoxon rank sum test was used for median values comparison. The relationship between SNRo and CD was tested with Pearson correlation coefficients.

The performance of the filtering method was expressed as sensitivity and specificity. Sensitivity and specificity of ECG signals before and after filtering were compared with the classification results of artifact-free ECG signals using Chi-square test. A *P* value of 0.01 was considered significant.

## 3. Results

The average duration of CPR was 6.8 ± 3.2 minutes. A total of 624 segments were extracted and 61 segments were excluded according to the exclusion criteria. Finally, a total of 563 CC related segments, including 283 VF, 208 PEA, and 72 ASY, were obtained for the study. The amplitude of artifact-free ECG signals was 0.7 ± 0.6 mV for VF, 0.8 ± 0.6 mV for PEA, and 0.05 ± 0.04 mV for ASY. The amplitude of corrupted ECG signals was 2.1 ± 1.2 mV for VF, 1.9 ± 0.8 mV for PEA, and 1.0 ± 0.7 mV for ASY.

### 3.1. Relationship between CC and SNR

A total of 107 segments of PEA were used for SNR estimation because the numbers of QRS complex within the selected artifact-free and corrupted ECG signals were equal.  Table 1 shows the medians (25/75 percentiles) and minimum and maximum value of the estimated SNR based on annotated underlying rhythms. A relative lower SNRo was observed for VF compared with that of PEA (*P* < 0.001) and the SNRo of ASY was significantly lower than PEA and VF (*P* < 0.001). After filtering with the proposed method and high-pass filter, the SNRfs were greatly improved in all of the rhythms (*P* < 0.001).

**Figure 4:**
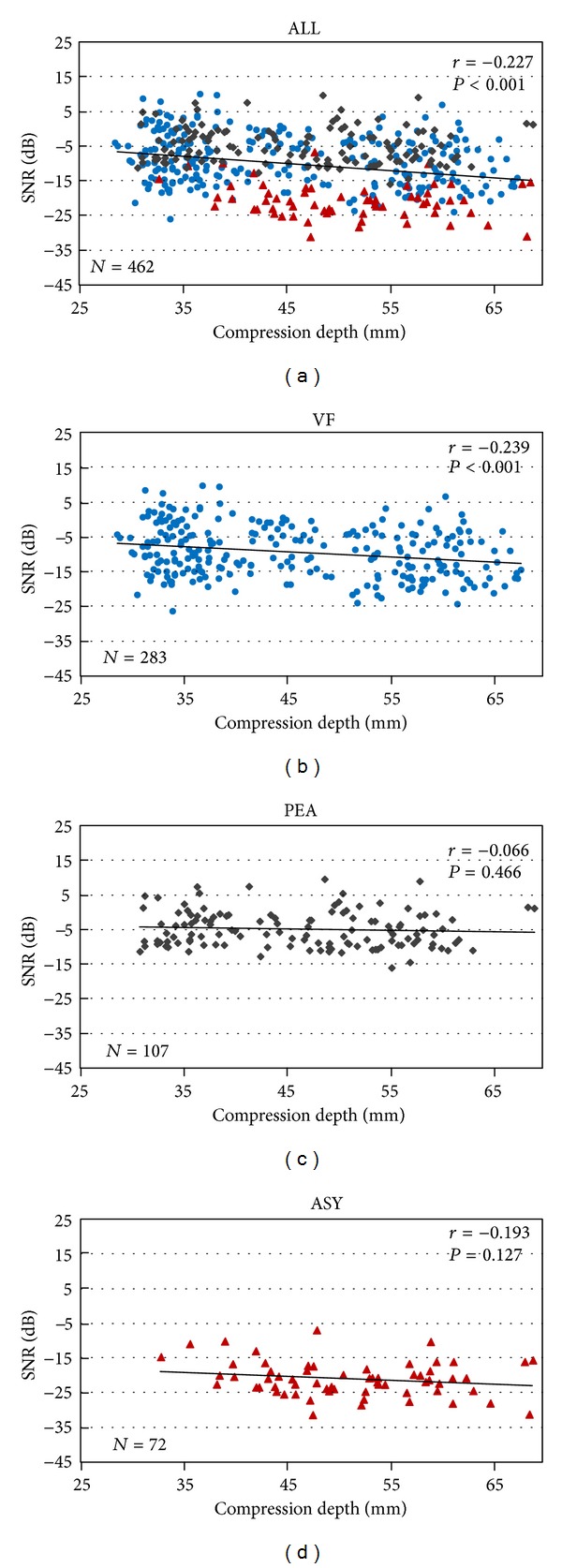
Linear regression results between SNRo and CD for the full database and different types of underlying rhythms (ventricular fibrillation, VF; pulseless electric activity, PEA; asystole, ASY).

The linear regression result between SNRo and CD is shown in Figure 4. The SNRo of the full database was negatively correlated with the CD (*r* = −0.227, *P* < 0.001). When each of the rhythms was investigated individually, negative correlation between CD and SNRo was only observed in VF (*r* = −0.239 and *P* < 0.001).

### 3.2. Performance of the Enhanced Adaptive Filtering Method

Table 2 shows the rhythm classification results for the artifact-free, CPR corrupted, and filtered signals with the use of PSR. The sensitivity and specificity were 99.0% and 98.2% for artifact-free signal. However, the specificity decreased to 46.8% and the sensitivity increased to 99.3% when the ECG signals were corrupted by CPR. After filtering by enhanced adaptive filter and high-pass filter, a sensitivity of 93.3% and 93.0% and a specificity of 96.0% and 80.4% were achieved.

## 4. Discussion

The present study confirmed that the SNRo of CPR corrupted ECG was negatively correlated with CD in a porcine model of prolonged cardiac arrest and CPR. Based on this observation, we developed an enhanced adaptive filtering method to suppress the CC-artifact by estimating the proportion of artifact within the corrupted ECG signal. The experimental results demonstrated that the enhanced adaptive filtering method could effectively reduce the residual component of artifact and improve the SNR of the ECG signal as well as the outcome of specificity.

### 4.1. Relationship between CC and SNR

The CC-artifact was predominant from the electrode-skin interface and generated by the contraction of thoracic muscles with direct impact of the compressions on chest wall [31]. Therefore, it was anticipated that deeper compression would cause more chest movements and introduced severe artifact to the ECG. In this animal study, we demonstrated that the SNRo of CPR corrupted ECG signals was negatively related to CD. However, when each of the rhythm was investigated, the SNRo was significantly lower for ASY compared to PEA and VF and the negative correlation between CD and SNRo was only observed in VF. For VF, the signal energy homogeneously distributed among all VF segments, and the value of SNRo was therefore correlated with the value of CD. For PEA, the energy of underlying signal depended on the number of QRS complexes appearing within a segment and might impact the correlation between SNRo and CD. For ASY, the energy of underlying signal is theoretically nearly 0 so that the SNRo should be –∞. However, randomized noisy signal and power supply artifact, together with artifacts produced by the amplifier and A/D converter, were introduced during measurement. Even though a band-pass filter was applied before analysis, the irregular residual signals within underlying ASY might still affect the value of signal energy and lead to insignificant correlation between SNRo and CD.

Compared with the result that was reported by de Gauna et al. [27], a relatively lower SNRo was observed in our study. The inconsistence may relate to the increased CD recommendation of the latest guidelines, which require a minimum of 50 mm in CD to ensure high quality CPR [10, 32]. At the same time, signal characteristics of porcine ECG such as amplitude and frequency might be different from that of human. The resulted SNRo thus would be affected by the spectral energy calculated from signal amplitude and frequency.

### 4.2. Improved Performance for the Enhanced Adaptive Filtering Method

Based on the findings that SNRo was negatively correlated with CD, we developed an enhanced adaptive filtering method to suppress the CPR artifact by estimating the proportion of artifact with the use of TTI as reference. Compared with the corrupted signal, both traditional fixed coefficient high-pass filter and proposed method could greatly improve the SNR and specificity. But compared with a specificity of 80.4% for high-pass filter, a remarkable improvement was achieved for the proposed method with a value of 96.0%.

The following modification in removing the CPR related artifact might contribute to the improved performance of the proposed method. Firstly, a parameter was introduced to estimate the proportion of artifact from PSD of ECG signal with the use of compression frequency as reference. The proportion of artifact was correlated with the power of artifact and therefore the SNR level. Secondly, the step size of commonly used LMS adaptive filter was dynamically adjusted by referenced TTI signal and the estimated proportion of artifact. This modification provided greater stability and convergence speed compared with traditional LMS based adaptive method which was used by Irusta et al. [17] and Aramendi et al. [18]. Therefore, the specificity of the proposed method was greatly improved compared with their results even though similar reference signals were used in both studies. Thirdly, the proportion of artifact was also used as an indicator to assess the artifact level in the filtered signal and to control the filtering iteration. This process was terminated only if the artifact level decreased to a predefined threshold. Compared with the MC-RAMP method which took use of several kinds of reference signals proposed by Husøy et al. [33] and Eilevstjønn et al. [20], the residual component of artifact could be further suppressed and the reliability for detecting a nonshockable rhythm was markedly improved.

Besides the enhanced adaptive filter, the algorithm used for rhythm classification also contributed to the improved specificity. The parameters were optimized according to clean ECG signals recorded from the animals when SPR was used [29]. Firstly, the ratio *r*′ was adjusted by the maximum number of data points visited in the box. This adjustment enlarged the difference between VF and PEA. Secondly, both window size and delay time were optimized when the phase space diagram was reconstructed. Consequently, the threshold of *r*′ increased from 0.15 to 0.24 for the detection of VF.

Although the SNR and specificity were greatly improved after filtering, the sensitivity decreased from 99.3% to 93.3%. It is because the enhanced filtering method also suppressed the component of underlying ECG signals while removing the CPR related artifact. As a result, amplitude of fine VF might be reduced to a level that is below the criteria for classification. When the nonshockable rhythms were investigated separately, the specificity for detecting ASY was relatively lower compared with that of PEA and still below the 95% limit recommended by AHA task force on AEDs [22]. This was consistent with the observation that CPR artifact suppression was particularly difficult in ASY [34, 35]. Yet, the 91.7% specificity for detecting ASY was still superior to reported results and the adverse effects of interruption of CC are likely to override the decrease in correctly detecting ASY.

### 4.3. Limitations

There are limitations that need to be acknowledged and addressed regarding the present study. Firstly, although the SNRo of CPR corrupted ECG was demonstrated to be negatively correlated with CD for the full database, this correlation was only observed in VF when different ECG rhythms were investigated individually. Additionally, the anatomy structure of human chest was different with that of the animals. Therefore the relationship between artifact level and CD in human beings at different underlying rhythms is still needed to be investigated. Secondly, only TTI signal was used as reference in this study; the effects of different reference signals on the performance of the proposed method have not been investigated. Thirdly, although a great improvement in specificity was achieved in this experimental trial, characteristics of ECG waveform, together with the CPR related artifact, may differ from the data that are recorded from patients who experienced out-of-hospital cardiac arrest and CPR. Performance of the proposed method therefore needs further clinical validating studies. Finally, even though the specificity for detecting a nonshockable rhythm was greatly improved and above the 95% limit recommended by the AHA task force on AEDs [22], the accuracy for detecting ASY was still low. Further studies that focused on the suppressing artifact of ASY, as well as the classification between ASY and VF, still need to be conducted.

## 5. Conclusion

This experimental animal trial demonstrated that the SNRo of ECG signal corrupted by CPR artifact was negatively correlated with CD and the enhanced adaptive filtering method could significantly improve the detection of nonshockable rhythms without compromising the ability to detect a shockable rhythm during uninterrupted CPR.

## Figures and Tables

**Table 1:** Estimated signal-to-noise ratio (SNR) for pulseless electrical activity (PEA), ventricular fibrillation (VF), and asystole (ASY) before and after filtering.

	Unfiltered	Adaptive filter	High-pass filter
Medians (dB) (25/75 percentiles)			
VF	−9.3 (−14.9/−3.6)^△△^	0.2 (−5.1/4.5)**	0.1 (−4.2/0.9)**
PEA	−6.2 (−9.0/−1.12)^△△^	0.1 (−3.6/3.4)**	−2.0 (−7.4/−0.6)**
ASY	−21.2 (−24.2/−18.5)^△△^	−12.7 (−15.0/−4.4)**	−7.1 (−10.7/−6.3)**
Range (dB) (min./max.)			
VF	−26.1/9.6	−18.2/20.0	−19.7/20.4
PEA	−16.0/9.9	−7.6/19.9	−14.0/14.7
ASY	−31.6/−10.0	−20.6/2.4	−18.4/1.7

**Compared with unfiltered signal, *P* < 0.001; ^△△^comparison among rhythm types, *P* < 0.001.

**Table 2:** Sensitivity and specificity for the artifact-free ECG and CC corrupted signals before and after filtering.

	Rhythm	Number	Artifact-free	Unfiltered	Adaptive	High-pass
Shockable (sensitivity)	VF	283	99.0%	99.3%	93.3%**	93.0%**

Nonshockable (specificity)	All	280	98.2%**	46.8%	96.0%^∗∗##^	80.4%**
	PEA	208	98.6%**	53.9%	97.6%^∗∗##^	86.3%**
	ASY	72	97.2%**	26.4%	91.7%^∗∗##^	63.9%**

**Compared with unfiltered signals, *P* < 0.001 and ^##^compared with high-pass filter, *P* < 0.001. VF: ventricular fibrillation, PEA: pulseless electrical activity, and ASY: asystole.
